# Computer-Based Diagnostic Expert Systems in Rheumatology: Where Do We Stand in 2014?

**DOI:** 10.1155/2014/672714

**Published:** 2014-07-08

**Authors:** Hannes Alder, Beat A. Michel, Christian Marx, Giorgio Tamborrini, Thomas Langenegger, Pius Bruehlmann, Johann Steurer, Lukas M. Wildi

**Affiliations:** ^1^Department of Rheumatology, University Hospital Zurich, Gloriastrasse 25, 8091 Zurich, Switzerland; ^2^Department of Rheumatology, Bethesda Hospital, Gellertstrasse 144, 4020 Basel, Switzerland; ^3^Department of Rheumatology, Zuger Kantonsspital, Landhausstrasse 11, 6340 Baar, Switzerland; ^4^Horten Centre for Patient Oriented Research and Knowledge Transfer, University of Zurich, Pestalozzistraße 24, 8091 Zurich, Switzerland

## Abstract

*Background*. The early detection of rheumatic diseases and the treatment to target have become of utmost importance to control the disease and improve its prognosis. However, establishing a diagnosis in early stages is challenging as many diseases initially present with similar symptoms and signs. Expert systems are computer programs designed to support the human decision making and have been developed in almost every field of medicine. *Methods*. This review focuses on the developments in the field of rheumatology to give a comprehensive insight. Medline, Embase, and Cochrane Library were searched. *Results*. Reports of 25 expert systems with different design and field of application were found. The performance of 19 of the identified expert systems was evaluated. The proportion of correctly diagnosed cases was between 43.1 and 99.9%. Sensitivity and specificity ranged from 62 to 100 and 88 to 98%, respectively. *Conclusions*. Promising diagnostic expert systems with moderate to excellent performance were identified. The validation process was in general underappreciated. None of the systems, however, seemed to have succeeded in daily practice. This review identifies optimal characteristics to increase the survival rate of expert systems and may serve as valuable information for future developments in the field.

## 1. Introduction

**Figure 1:**
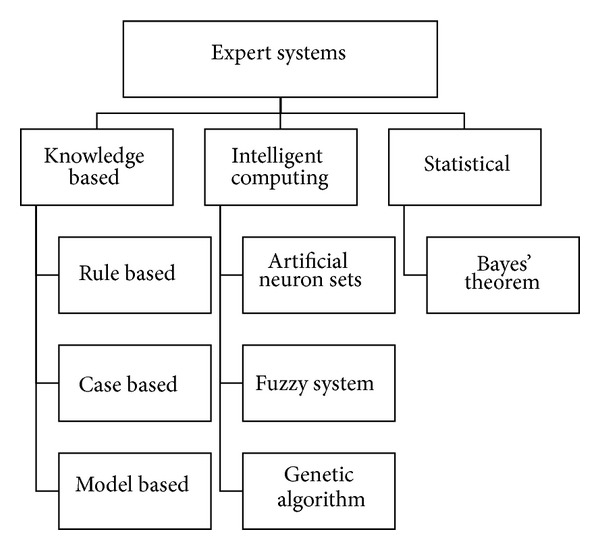
Common methodologies for expert systems.

**Figure 2:**
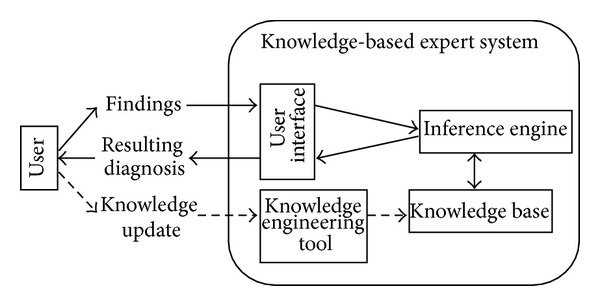
Typical structure of a knowledge-based expert system. Based on Buchanan [3], the user interface allows the nonexpert to enter the symptoms and findings [3] and presents the diagnostic output. The knowledge base provides the knowledge. Different ways of representation, such as rules, models, or cases, can be chosen. The inference engine examines the knowledge base and produces reasoning [15]. The knowledge engineering tool allows for changing or enlarging the knowledge base by adding further rules, cases, or models [7]. There may also be an explaining component, which illustrates the diagnostic process and which gives a rationale [7]. A knowledge-based expert system with an empty knowledge base is called shell. It can be used for the development of other expert systems by adding a new knowledge base [7].

Rheumatologic diseases manifest themselves in varying combinations of symptoms and signs, particularly at early stages, and therefore make differential diagnosis a challenge, especially for nonrheumatologists including general practitioners. Since diagnosis at an early stage and adequate treatment improve prognosis, assistance in establishing diagnosis is desirable. Given the substantial progress in computer science in the last years, the idea of computers taking the role of diagnostic support is not far-fetched. Software applications have affected decision processes in clinical routine, for example, in controlling depth of anesthesia [1] or in detecting drug interactions [2]. Software tools to support physicians in the diagnostic process have been developed in almost every field of medicine. A widely utilized type is the so-called expert system, defined as artificial intelligence program designed to provide expert-level solutions to complex problems [3]. Figures 1 and 2 give an overview of the concept.

Pandey and Mishra distinguished between knowledge-based systems and intelligent computing systems [4]. There are three different approaches to knowledge-based systems depending on the form of knowledge representation: rule based, case based, and model based. In rule based reasoning, the knowledge is expressed by rules, often IF*⋯* THEN*⋯* rules [4]. The rules can be newly developed or can be extracted from decision tables or decision trees [5]. In case of based reasoning, the inference engine searches the knowledge base for similar cases. Finally, models, that is, biochemical or biophysical, can also form the knowledge [4]. The typical knowledge-based expert system consists of four parts.  Figure 2 illustrates its structure.

The approaches to intelligent computing systems are artificial neuron nets, genetic algorithm, and fuzzy systems. Artificial neuron nets are built like biologic intelligent nervous systems and are regarded as learn-like [4]. Individual variables receive inhibitory and excitatory inputs like neurons. The calculations are made in parallel, not only sequentially like in other methodologies [6]. Genetic algorithm mimics the process of natural evolution and is mainly used in search processes. Fuzzy systems are usually based on rules, but the reasoning is approximate to cope with uncertainty and imprecision because the rules are given varying truth-value using fuzzy sets [7]. Thus, linguistic certainty or frequency levels, such as probable or seldom, derived from medical texts or experts can be incorporated into the knowledge base [8].

Bayes' theorem is a statistical method. The probability of a diagnosis is calculated with the accuracy of a test or a clinical finding and the prevalence of the disease [9]. Thus Bayes' theorem sets probabilistic values for each diagnostic output [4].

Different methodologies are often combined, which are then called hybrid expert systems [10].

Already in 1959, Ledley and Lusted anticipated the use of computers in supporting decisions and proposed different mathematical models to emulate the reasoning in medical diagnosis [11]. Since then, the number of expert systems in medicine has grown rapidly. The first expert systems were developed in the 1970's. Two well-known pioneer expert systems are MYCIN and INTERNIST-1. They were archetypes for following expert systems, but they also demonstrated the challenges in the development of such tools. MYCIN was developed at the Stanford University in the 1970s. It was used for diagnosis and therapy of bacterial infection and has become the probably best-known expert system in medicine [3]. INTERNIST-1 was developed at the University of Pittsburgh [12]. It was designed to assist physicians in the diagnosis of complex and multiple diseases in internal medicine covering more than five hundred diseases [13]. The problems encountered in developing INTERNIST-1 and its successors showed that a comprehensive knowledge base is needed for a correct diagnosis of complex diseases in internal medicine [7].

Several somewhat outdated review articles explored the development and application of expert systems in medicine in general [4, 10, 14]. In 1991, Bernelot Moens and van der Korst reviewed the literature assessing computer-assisted diagnosis of rheumatic diseases [15]. Meanwhile, also in rheumatology new expert systems have emerged and earlier expert systems have been improved to meet the many demands of modern rheumatology: establishing an early diagnosis with the highest probability to allow for a better outcome with the help of a prompt treatment.

Besides an overview of characteristics, comprehensiveness, and validation of existing diagnostic expert systems in rheumatology, this systematic review seeks to point out whether the current expert systems fulfill the expectations of clinicians in daily practice and finally what the characteristics of an optimal system would be.

## 2. Methods

The systematic literature review was carried out following the PRISMA statement [16]. No ethics board approval or consent of any individual was necessary. The research questions were as follows: what information is currently available on diagnostic expert systems in rheumatology, how do these systems work, what is their validity and their applicability in daily practice, and finally what is an optimal diagnostic expert system expected to be.

### 2.1. Scenarios

In the optimal scenario we anticipated to find comprehensive reports on each individual diagnostic expert system including information on the precise diagnostic algorithm, the targeted diseases, a well-described validation cohort, and predictive values for diagnostic performance. The data would allow for a statistical comparison of the expert systems. In a suboptimal scenario, only descriptive reports of expert systems will be found allowing for a comprehensive overview of the past developments without statistical comparability.

### 2.2. Systematic Literature Search

Medline, Embase, and Cochrane Library were searched using the following Medical Subject Heading (MESH) terms: “rheumatic diseases,” “rheumatology,” “arthritis,” “computer assisted diagnosis,” and “expert systems.” No restrictions were placed on publication date. Only literature in English or German was considered. The last search was run on February 10, 2014.

### 2.3. Selection of Articles

**Figure 3:**
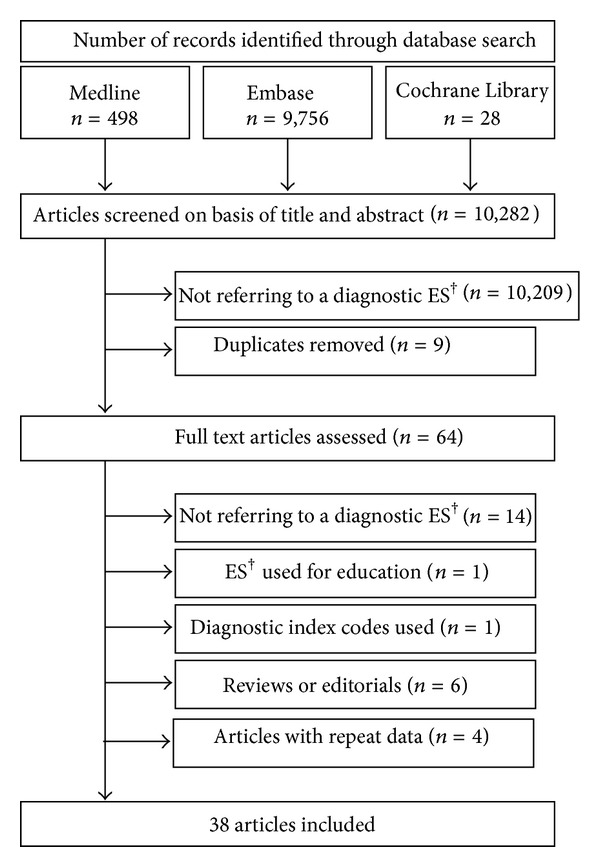
Selection of publications.

The literature was screened based on title and abstract of the records. All publications referring to diagnostic expert systems in rheumatology or in a rheumatic subfield were included. Reviews, editorials, and literature which described an expert system only used for education of healthcare providers and therefore not used in diagnostics were excluded. Also, literature that referred to an expert system used for identifying solely the stage of a disease and hence not used for diagnosing a disease itself was excluded. Records which described an expert system applied only to image analysis were not considered either. Literature referring to data mining strategies using index diagnoses or solely epidemiological variables was excluded as well.  Figure 3 shows a flow diagram of the selection of studies. In case of uncertainties, inclusion or exclusion was based on consensus.

### 2.4. Data Extraction and Statistical Analysis

Year of the last update of the system, number of considered rheumatic diseases, targeted diseases, information to feed the expert systems (history, clinical exam, laboratory analyses, and imaging studies), methodology of the inference mechanism, and embedding of accepted disease criteria sets such as the American College of Rheumatology (ACR) or The European League Against Rheumatism (EULAR) criteria were extracted using standard forms.

For the description of the validation method and the performance, the following information was extracted from the articles: number of cases used for the validation, determination of the resulting diagnosis, identification of the correct diagnosis, the reference diagnosis, percentage of correctly identified cases, sensitivity and specificity, positive predictive values, negative predictive values, positive likelihood ratio, and negative likelihood ratio.

Only descriptive statistics are reported. Statistical analyses could not be performed due to the lack of information.

## 3. Results

### 3.1. Literature Searches

A total of 10,282 references were identified using the search strategy. Seventy-three articles related to diagnostic expert systems in rheumatology were included. Nine duplicates were excluded. The remaining 64 full text articles were then assessed. One record describing an expert system developed solely for education [17] and one record referring to an expert system that was not designed for clinical use [18] were excluded. Six reviews or editorials were excluded [6, 15, 19–22]. In the case of repeated reports, either the original or the more comprehensive article was included in the evaluation leading to a final number of 38 original articles (Figure 3). In these 38 articles, 25 different expert systems and their successors or further developments are presented. Most of the articles shown in this review presented the development and the methodology of expert systems.

### 3.2. Characteristics of the Identified Expert Systems

Table 1 gives an overview over the 25 identified expert systems and their characteristics. The number of considered diseases varies from one to 170. Both the amount and the nature of information to feed the expert systems vary according to the targeted disease group and inference mechanisms. The following methodologies of expert systems were observed: rule based, case based, model based, artificial neuron nets, fuzzy systems, Bayes' theorem, and other not further described algorithms or calculation tools (Figure 1). Rule-based systems were the most frequent. Twelve different spectra of targeted diseases were found. Six expert systems used ACR or EULAR criteria to establish a diagnosis.

### 3.3. Validation

Table 2 summarizes the validation of the expert systems. 19 of the 25 expert systems (76%) were validated. The number of cases used for the validation varied widely between 32 real cases and 12 000 simulated patients. Different units of measurement were selected to report the performance of the expert systems, mostly the percentage of correctly diagnosed cases, sensitivity and specificity. The proportion of correctly diagnosed cases—the diagnostic accuracy—was between 43.1 and 99.9%. Values for sensitivity and specificity ranged from 62 to 100, and 88 to 98%, respectively. Positive or negative predictive values and likelihood ratios were only surveyed for two expert systems [26, 27]. Liu et al. reached a positive predictive value of 91%. Binder et al. showed a positive likelihood ratio of 12.1 (95% CI 7.70–19.1) and a negative likelihood ratio of 0.187 (95% CI 0.099–0.351). Excluding this last report, confidence intervals were not indicated.

The reference standards were chosen differently: diagnoses according to established criteria, consensus diagnoses, discharge diagnoses, and diagnoses provided by a rheumatologist were used most often as reference. Three expert systems presented certain criteria for the determination of the resulting diagnosis when several diagnoses were presented as a result or when a probability value was added to the diagnosis.  Table 3 presents the chosen reference diagnoses and the determinations of the resulting diagnoses.

An article that reports on the applicability of a rheumatological expert system in clinical routine could not be identified in the published literature.

## 4. Discussion

The main result of this systematic review is threefold. First, an overview over 25 different diagnostic expert systems designed for rheumatology is given. Second, it is shown that the different designs and validation methods of the expert systems hinder the comparison of their performances. Third, we found no publications reporting on the routine application of an expert system in rheumatology.

Artificial intelligence has achieved enormous progress in its development and computers have outclassed human beings in various fields, such as computer chess or IBM's Watson winning on the quiz show “Jeopardy!” Given this progress in technology and the time period covered by this systematic review of over forty years, the low number of identified expert system is surprising. The reasons would be either low interest in supportive software or, more likely, the difficulties encountered in simulating the complex human diagnostic process. Spreckelsen et al. [60] reported that developers of knowledge-based systems regarded pharmacovigilance, intensive care monitoring, and support for guidelines and clinical pathways as the most promising fields of knowledge-based systems. In other words, systems covering clearly defined decision rules or comparing databases. Diagnostic support was less favorably judged. In rheumatology in particular, the diagnostic process is hampered by multiple factors. First of all, nonspecific findings occurring in multiple rheumatic diseases are common and consequently complicate the knowledge representation in expert systems. Second, there is a lack of epidemiological data concerning the prevalence and incidence of rheumatic diseases as well as sensitivity and the specificity of single findings in diseases. Third, even if available for a large population, such data vary greatly amongst ethnic groups and regions becoming an increasing problem in times of global migration. Fourth, for many of the disease-specific findings, there are no internationally established standardized cut-off values. And finally, many rheumatic diseases can coexist with each other in overlap syndromes.

Nevertheless, the growing understanding of diseases and the corresponding findings or symptoms will facilitate the representation of medical knowledge and decision processes in the future.

### 4.1. Validation of Expert Systems

In consequence of the variation in the method of validation, the achieved validation results could not be compared with each other. The reason for this variability probably lies in two elements.

First, the result of the expert systems to be compared with the reference diagnosis was presented in different ways. Some expert systems indicated a probability value of the calculated resulting diagnosis, and others present a hypotheses list. Final diagnoses in rheumatology often remain descriptive or incomplete and evolve over time as many of the rheumatic disorders present atypically and do not completely fulfill a diagnostic criteria set at the beginning. This issue is met by the presentation of the results as a hypotheses list or probability values, which can, as an important advantage, multiply the user's own differential diagnosis and lead to more focused testing. Yet, this method causes difficulties in the validation and the comparison of expert systems. For example, the diagnostic accuracy is erroneously high if a diagnosis at a low position in the hypotheses list or a diagnosis with a low probability value is accepted as a correct resulting diagnosis during the validation process.

Second, there is a lack of widely accepted reference standards for the correct diagnosis to compare the resulting diagnosis with. Some authors used diagnoses in medical records or discharge diagnoses as a comparator assuming the correctness of their peers, some chose the consensus of rheumatologists, and others used diagnoses according to official diagnostic criteria sets. The latter is probably the most reliable way; however, even if international consensus criteria exist, there are still many different criteria sets especially for rare diseases where the superiority of one set over the other and in particular the threshold for a diagnosis remains a matter of debate. In addition, many of these criteria sets were established to obtain homogenous cohorts in clinical trials leading to a low sensitivity in early or mild disease.

Another approach was the assessment of the interobserver variability by Hernandez et al. [34] and Martín-Baranera et al. [35]. Here, the distance between the resulting diagnoses of clinicians and RENOIR was calculated without setting a reference diagnosis. By this means the uncertainty of the final diagnosis and the error proneness of clinicians were taken into account.

The transferability of expert systems to the general population (the external validity) can be tested with a validation in a developer-independent clinical setting. Only AI/RHEUM, CADIAG, and RHEUMA [29, 39, 40, 49] were validated this way, resulting in a lack of data on the transferability to daily practice of most of the presently available expert systems.

### 4.2. Clinical Use and Requirements of Expert Systems in Practice

Besides the internal and external validity, the following features are, according to Kawamoto et al., highly associated with an expert system's ability to improve clinical practice: the availability at the time and location of decision making, the integration into clinical workflow, and the provision of recommendations rather than a pure assessment [61]. The wider use of computers in clinical routine, such as the possible use of tablet computers on ward rounds, will facilitate the integration into clinical workflow and enhance the availability at the time and location of decision making. The need of more detailed documentation for quality assurance may have a positive influence as well. Boegl et al. are the only authors who reported the clinical use of their diagnostic expert system Cadiag-4/Rheuma-Radio. The expert system was incorporated in the medical information system of the respective clinic [30]. For the lack of accessibility of diagnostic support, the universally present search engines for the World Wide Web have become a popular alternative with an astonishing accuracy as shown by Tang and Ng [62] and Lombardi et al. [63].

Kolarz et al. [37] and Schewe and Schreiber [49] regarded the time required for data input as the most limiting factor. Considering the smaller amount of input data and consequently the shorter input time, specialized and restricted expert systems like the laboratory results analyzing system presented by Binder et al. [26] have the edge over more comprehensive systems. Kaplan [41] presented a system with a provisional hypothesis list, which updates after every further input. Here, the data input is limited; hence, there is a risk of missed diagnoses due to the less thorough questioning. The required time for data input would decrease if the expert system was compatible with the institutional medical information system and consequently could allow direct access to all electronically stored patient data comprising patient history, physical exam, imaging studies, and laboratory analyses. The latter include the increasingly important biomarkers [64, 65]. Then again the data input and the required time depend on an intuitive user interface, which Boegl et al. believed to have the biggest influence on the clinical success [30].

The reason for the absence of expert systems in clinical use hitherto has been discussed in detail in the literature. Mandl and Kohane claimed that health information technology in general was in arrears compared to other industries. Also they took the health information technology products as too specific and incompatible with each other [66]. Spreckelsen et al. evaluated an online survey of researchers and developers of knowledge-based systems. They stated that the lack of acceptance by the medical staff is the main problem in the application of knowledge-based systems in medicine [60]. The different points of view of developers and clinicians show that a better cooperation is necessary. Expert systems have to be adapted to clinical problems and to clinical workflow. On the other hand, clinicians should become more aware of the supportive possibilities of expert systems.

### 4.3. Importance of Targeted User Group

In spite of computerized assistance, the user of the expert system needs rheumatologic fundamentals for the detection and the correct description of rheumatologic findings. CADIAG, AI/RHEUM, RENOIR, RHEUMexpert, and MESICAR were specifically developed for the assistance of nonrheumatologists [31, 37, 39, 48, 50]. These systems were designed to remind the nonspecialist of rare diseases or to indicate the cases which needed immediate treatment. Yet, an expert system's outcome highly depends on the entry of correct parameters. Therefore, educational parts were added to some of the expert systems to increase the user's diagnostic skills. These educational parts explain certain symptoms or show photographs of findings [30, 42, 51]. Also, some systems provided a link to literature, such as Medline, for further information [30, 42]. The AI/RHEUM and CADIAG project presented the most extensive educational parts. A widely accepted system ideally covers the demands of generalists and specialists offering an easy understandable handling and not being too basic at the same time.

### 4.4. Diagnostic Criteria Sets

The integration of widely accepted diagnostic criteria sets such as the ACR or EULAR criteria into the diagnostic process would increase the acceptance and credibility of an expert system. It also reduces the influence of individual diagnostic strategies of the developers. Nevertheless, only six of the identified expert systems reported the integration of such criteria sets into their expert database. The downside of diagnostic criteria originating primarily from classification criteria for the inclusion into clinical trials, however, is the generally low sensitivity in early disease. This insensitivity of some criteria, such as the 1987 ARA criteria for rheumatoid arthritis, forced Leitich et al. to modify the criteria using fuzzy sets to gain different levels of sensitivity [32]. The recent development of official diagnostic criteria, which are more dedicated to the diagnosis in an early stage of the disease [67], will make their use in the design of expert systems more attractive. Furthermore, some methodologies are ill suited to the use of diagnostic criteria, such as a mere probabilistic approach like Bayes' theorem or artificial neural network. These systems extract their knowledge base from patient data, such as symptoms and clinical findings, and the corresponding diagnoses assuming a correctness of the chosen diagnosis. Diagnostic criteria cannot be included in these systems without the combination with another methodology or an adaption of the reasoning process like the review of symptom weighing. Other ways of knowledge representation facilitate the usage of official diagnostic criteria, like rule-based reasoning though the minority of the articles presenting a rule-based expert system reported an integration of official diagnostic criteria.

### 4.5. Limitations

Although a thorough systematic search has been performed in the most relevant databases, some reports could have been missed if written in other languages than English or German. As most of the current literature is published in English at least as an abstract, we are confident that we did not miss relevant articles on diagnostic expert systems in rheumatology. The number of expert systems which have remained unpublished because of their expected commercial use or the abortion of the system at an early stage is hard to estimate.

The reported expert systems showed a great variety in diseases spectrum, methodology, and validation status. This made a statistical comparison of the systems impossible.

And finally, the important topic of patient reported outcomes which are of increasing importance not only in clinical trials and patient's follow-up but also in the diagnostic process was beyond the scope of this review.

## 5. Conclusion

In conclusion, this systematic review shows that the many attempts made for an ideal expert system in rheumatology in the past decades have not yet resulted in convincing validated tools allowing for reliable application in daily practice. Nevertheless, the demand in support by expert systems is pressing as the knowledge about the rheumatic diseases increases and the therapeutic options especially in early disease stages are growing constantly. An ideal diagnostic expert system in rheumatology would have the following characteristics. The expert system would allow for universal integration into the clinical workflow as well as rapid and intuitive data input. Since rheumatologic diagnoses cannot always be definite, the resulting diagnosis would have a probabilistic grade to indicate uncertainty. The system would also have an educational component to improve the nonexpert's ability to recognize pathological findings. Finally, accepted diagnostic criteria sets would be applied to increase the general validity of the system's diagnostic process.

Based on the demand of such a tool and the progress made hitherto it seems to be a matter of time until new and promising expert systems enter clinical practice.

## Figures and Tables

**Table 1:** Characteristics of the identified expert systems.

Name of ES^a^ or first author	Year of last update	Number of diseases	Targeted diseases	Input for ES^a^	Methodology	Reference
Romano	2009	2	Prosthesis infection	L^b^, I^c^	Calculation tool	[23]
Watt	2008	1	Knee osteoarthritis	H^d^, E^e^, I^c^	Bayesian belief network	[24]
Provenzano	2007	3	Chronic pain	H^d^	Discriminant analysis	[25]
Binder	2005	5	Connective tissue diseases	L^b^	Case based reasoning	[26]
Liu^f^	2004	1	RA^g^	H^d^, L^b^	Algorithm	[27]
Lim	2002	24	Arthritic diseases		Hierarchical fuzzy inference	[28]
CADIAG^f^	2001	170	Rheumatic diseases	H^d^, E^e^, L^b^, I^c^	Rule based, fuzzy sets	[8, 29–33]
RENOIR^f^	2001	37	Rheumatic diseases	H^d^, E^e^, L^b^, I^c^	Rule based, fuzzy sets	[34–36]
RHEUMexpert	1999		Rheumatic diseases	H^d^, E^e^, L^b^, I^c^	Rule based	[37]
Zupan	1998	8	Rheumatic diseases	H^d^	Rule based	[38]
AI/RHEUM	1998	59	Rheumatic diseases	H^d^, E^e^, L^b^, I^c^	Rule based	[39–43]
Dzeroski	1996	8	Rheumatic diseases	H^d^	Rule based and statistical	[44]
Heller^f^	1995	6	Vasculitis	H^d^, E^e^, L^b^	Bayesian classifier	[45]
Astion	1994	1	Giant cell arteritis	H^d^, E^e^, L^b^	Neural networks	[46]
Barreto	1993	2	RA^g^ and SLE^h^	H^d^, E^e^, L^b^, I^c^	Neural networks, fuzzy sets	[47]
MESICAR	1993		Rheumatic diseases		Model based	[48]
RHEUMA	1993	67	Rheumatic diseases	H^d^, E^e^, L^b^, I^c^	Rule based	[49]
Bernelot Moens	1992	15	Rheumatic diseases	H^d^, E^e^, L^b^, I^c^	Bayes' Theorem	[50–52]
Sereni	1991	1	Temporal arteritis	H^d^, E^e^, L^b^	Bayes' Theorem, decision tree	[53]
Rigby	1991	1	RA^g^	H^d^, E^e^	Bayesian and logistic regression	[54]
Schewe^f^	1990	32	Knee pain	H^d^	Rule based	[55]
Prust	1986	2	Ankylosing spondylitis and SLE^h^	H^d^, E^e^	Scoring tool	[56]
Gini	1980	7	Arthritic diseases	H^d^	Rule based	[57]
Dostál	1972	1	RA^g^	H^d^	Bayes' Theorem	[58]
Fries	1970	35	Arthritic diseases	H^d^	Statistical	[59]

^a^ES: xpert system, ^b^L: laboratory results, ^c^I: imaging results, ^d^M: medical history, ^e^E: physical examination, ^f^ACR or EULAR criteria included, ^g^RA: rheumatoid arthritis, ^h^SLE: systemic lupus erythematosus.

**Table 2:** Validation of the identified expert systems.

Name of ES^a^ or first author	Number of cases used for validation	Percentage of diagnoses correct	Sensitivity	Specificity	Reference
Romano	32				[23]
Watt	200	100%			[24]
Provenzano	511	22.9–69.7%^b^			[25]
Binder	325		82.6% CI^c^: 68.0–91.7	93.2% CI^c^: 89.4–95.7	[26]
Liu	90	95%	100%	88%	[27]
Lim	No validation				[28]
CADIAG^d^	54	48%^e^			[29]
RENOIR^d^	32	75%			[36]
RHEUMexpert	252		32–77%^f^	70–73%^f^	[37]
Zupan	462	46.8% SD^g^: 3.9			[38]
AI/RHEUM^d^	94	80%			[42]
Dzeroski	462	47.2–50.9%^b^			[44]
Heller	12000 computer simulated cases	84.15–99.9%^f^			[45]
Astion	807		94.4%	91.9%	[46]
Barreto	No validation				[47]
MESICAR	No validation				[48]
RHEUMA	51	89%^e^			[49]
Bernelot Moens^d^	570	76%/80%^b^ SE^h^: 10.2/9.5	62%	98%	[51]
Sereni	341				[53]
Rigby	No validation				[54]
Schewe	358	74.4%			[55]
Prust	No validation				[56]
Gini	No validation				[57]
Dostál	553	80%			[58]
Fries	190	76%			[59]

^a^Expert system, ^b^multiple formulas were applied, ^c^CI: 95% confidence interval, ^d^more than one evaluation, ^e^evaluated in other clinic than developed, ^f^results depending on disease, ^g^SD: standard deviation, ^h^SE: standard error.

**Table 3:** Reference diagnoses and the determinations of the resulting diagnoses.

Name of ES^†^ or first author	Reference diagnosis	Determination of the resulting diagnosis	Reference^§^
Watt	NIH Osteoarthritis initiative data base		[24]
Binder	Diagnosis according to established criteria		[26]
Liu	Consensus of rheumatologists		[27]
CADIAG	Discharge diagnosis	Among first 5 hypotheses	[29]
RENOIR	Discharge diagnosis		[36]
RHEUMexpert	Discharge diagnosis		[37]
AI/RHEUM	Initial diagnosis of a rheumatologist	At the possible level	[42]
Astion	Vasculitis database of the American College of Rheumatology		[46]
RHEUMA	Discharge diagnosis		[49]
Bernelot Moens	Outcome over time and consensus of rheumatologists		[51]
Sereni	Biopsy		[53]
Schewe		In the hypotheses list	[55]
Dostál	Diagnosis provided by a rheumatologist		[58]
Fries	Diagnosis provided by a rheumatologist		[59]

^†^ES: expert system, ^§^reference.
